# Reconstruction of Optical Vector-Fields With Applications in Endoscopic Imaging

**DOI:** 10.1109/TMI.2018.2875875

**Published:** 2018-10-12

**Authors:** Milana Gataric, George S. D. Gordon, Francesco Renna, Alberto Gil C. P. Ramos, Maria P. Alcolea, Sarah E. Bohndiek

**Affiliations:** 1 Department of Pure Mathematics and Mathematical StatisticsUniversity of Cambridge 2152 CambridgeCB3 0WBU.K.; 2 Department of EngineeringUniversity of Cambridge 2152 CambridgeCB3 0FAU.K.; 3 Instituto de Telecomunicações, Faculdade de Ciências da Universidade do Porto4169-007PortoPortugal; 4 Nokia Bell Labs 5676 CambridgeCB3 0FAU.K.; 5 Wellcome Trust-MRC Cambridge Stem Cell Institute, University of Cambridge 2152 CambridgeCB2 0XZU.K.; 6 Department of PhysicsCancer Research UK Cambridge Institute, University of Cambridge 2152 CambridgeCB3 0HEU.K.

**Keywords:** Inverse problem, image reconstruction, calibration, Fourier features, optical phase and polarization, endoscope

## Abstract

We introduce a framework for the reconstruction of the amplitude, phase, and polarization of an optical vector-field using measurements acquired by an imaging device characterized by an integral transform with an unknown spatially variant kernel. By incorporating effective regularization terms, this new approach is able to recover an optical vector-field with respect to an arbitrary representation system, which may be different from the one used for device calibration. In particular, it enables the recovery of an optical vector-field with respect to a Fourier basis, which is shown to yield indicative features of increased scattering associated with tissue abnormalities. We demonstrate the effectiveness of our approach using synthetic holographic images and biological tissue samples in an experimental setting, where the measurements of an optical vector-field are acquired by a multicore fiber endoscope, and observe that indeed the recovered Fourier coefficients are useful in distinguishing healthy tissues from tumors in early stages of oesophageal cancer.

## I. Introduction

Recently, there has been a significant interest in developing new types of optical fiber endoscopes for medical imaging applications [9], [13], [18], [30], [45]. Typically, these new endoscopes aim to be thinner, and therefore less invasive, and/or use different properties of light than conventional white light endoscopes making them more sensitive for detecting diseases such as cancer [50]. When a tissue is illuminated by light of high spatial and temporal coherence, a full optical vector-field reflected from the tissue consists of amplitude, phase and polarization information [2], [24], [36], [37], [41]. Phase and polarization have recently shown promise as diagnostic indicators, but are discarded by conventional white light endoscopes which record amplitude information only. Phase is highly sensitive to surface scattering that arises due to microstructural tissue changes in early cancer, creating distorted reflected wavefronts [7], [22], [44], [46], [47]. This effect has been utilized in phase contrast and quantitative phase microscopy to predict recurrence of prostate cancer [43]. Similarly, polarization information can indicate the formation of dense collagen networks [8], and the concentration of other polarization-sensitive compounds, such as glucose, linked with early cancer [4], [29]. This has found use in the diagnosis of colon [3], [33] and gastric cancers [48]. Currently, there are no commercial phase and polarization endoscopes but many prototype devices have been demonstrated [18], [34], [39], [45], [49].

To achieve phase and polarization imaging in fiber endoscopes, the underlying transformation of the optical fiber needs to be characterized. In realistic clinical settings, this transformation changes frequently due to bending and temperature fluctuations and it is therefore important that the characterization is efficient and accurate. For the characterization, typically a set of known fields that form some kind of a basis are input into one end of the fiber and the resulting outputs are recorded at the other end, a procedure termed *calibration*. The task then becomes to recover a representation of the optical field reflected from a tissue given the calibration measurements and the samples of the output field measured by an imaging sensor outside of the fiber.

In this paper, we investigate the following questions: (i) is there a particularly useful representation of the optical field reflected from a tissue that can be used for detecting optical aberrations associated with early cancer, and (ii) how can such a representation be recovered by an efficient and reliable algorithm from raw endoscopic measurements, i.e. from the calibration measurements and the samples of the output field?

To address these questions, we show that a Fourier representation recovered directly from the raw measurements has the statistical power to distinguish healthy tissues from tumors, and we provide a general reconstruction framework that can perform such recovery efficiently and stably.

More concretely, after reviewing related previous works in Section I-A, in Section II we introduce a general reconstruction framework for the recovery of a two-dimensional complex vector-field, where different regularization terms are permitted and the bases used for image representation and device calibration are allowed to be different and/or non-orthogonal. In Section III we demonstrate that it is possible to extract informative features for detecting cancer from images of simulated tissue samples by projecting them onto a Fourier basis and observing the decay of their respective Fourier coefficients. In Section IV, we apply our approach to experimental data acquired using a custom-built fiber endoscope [25] and recover synthetic holographic images as well as images of mouse oesophageal tissue containing small tumors (lesions). In particular, by recovering images of a biological tissue with respect to a Fourier basis using $\ell _{1}$-regularization, we observe that the corresponding Fourier coefficients are indicative of differences between lesions and healthy tissues and demonstrate their potential for medical diagnostic applications. We conclude with a discussion of our results and directions for future research in Section V.

### A. Relation to Previous Work

In imaging through optical fibers or other scattering media, typical recovery procedures use the same, finite-dimensional basis for calibration and image representation in conjunction with standard inversion techniques. They start by discretizing the mathematical operator of the fiber as a mapping between pixels at different ends of the fiber $\mathbf {A}:\mathbf {x}\in \mathbb {C}^{k} \mapsto \mathbf {y}\in \mathbb {C}^{n}$, leading to a transmission matrix $\mathbf {A}\in \mathbb {C}^{n\times k}$ which is then characterized through calibration. The calibration inputs are collected in the columns of matrix $\mathbf {X}_{cal}\in \mathbb {C}^{k\times m}$ and the corresponding outputs in the columns of matrix $\mathbf {Y}_{cal}\in \mathbb {C}^{n\times m}$. Most existing systems use a full orthogonal basis of the discretized input space as the calibration inputs, e.g. a set of tilted plane waves (a Fourier basis) [16] or a Hadamard basis generated using a phase-only spatial light modulator [35]. The orthogonality of such bases ensures that $\mathbf {X}_{cal}$ is unitary. Then, by assuming that $\mathbf {A}$ is also unitary, images can be recovered using *phase conjugation*. In this approach a (generalized) inverse of the transmission matrix is calculated as $\mathbf {X}_{cal}\mathbf {Y}_{cal}^{*}$, where $\cdot ^{*}$ denotes the conjugate transpose, and a representation of $\mathbf {x}$ with respect to the calibration inputs is recovered as $\mathbf {X}_{cal}\mathbf {Y}_{cal}^{*}\mathbf {y}$ [17], [18], [35]. Although simple and straightforward to compute, the unitary assumptions in this approach are typically violated in practice [14]. In the context of imaging through scattering media, the inversion of matrix $\mathbf {A}$ was also performed through alternative approaches to phase-conjugation such as least-squares or Tikhonov regularization [36], [37]. In particular, these applications do not explore $\ell _{1}$-regularization, which becomes a natural choice when reconstructing images with respect to a sparse basis. In this paper, we aim to reconstruct the real-world tissue images which are expected to be sparse in a basis such as Fourier, since these images are relatively smooth without abrupt discontinuities.

When compared to these conventional techniques, we emphasize that our new framework can recover a representation of the unknown optical field with respect to any particular infinite-dimensional basis which is allowed to be different from the one used for calibration, directly from the raw measurements. If an image representation with respect to a particular basis (such as Fourier) is desired, alternatively to our new approach one could in principle use the conventional techniques to recover an approximation to such a representation as we now describe. One could calibrate the fiber with respect to a Fourier basis and use standard techniques to reconstruct images with respect to the same basis. However, in high resolution imaging, calibration with respect to a Fourier basis may become prohibitively slow in practice and it may be preferable to use different, more efficient systems for calibration, as we do in this paper. Another possible approach is to first recover the image with respect to the calibration basis and then approximate its Fourier coefficients in a post-processing step. However, as a two-stage procedure, such approach is inherently less efficient and suffers from greater error than the approach proposed in this paper, which is able to recover Fourier coefficients directly from the raw measurements.

In the earlier work [25], an endoscope with a commercially available multicore fiber (MCF) bundle was developed to produce images of phase and polarization for early cancer detection. There, a set of calibration inputs was chosen to greatly speed up experimental measurement time. Specifically, a set of Gaussian-like spots translated in small steps was used to enable parallelized calibration by exploiting the localized confinement of light in the MCF structure. However, this input basis is non-orthogonal so phase-conjugation cannot be naively applied. Instead, a reconstruction algorithm which solves one inverse problem per pixel was used to recover the images in a pixel basis. In particular, representations with respect different bases were not considered. While preserving the benefits of efficient experimental calibration achieved with a system tailored to the fiber structure, by using the framework presented here, we are now able to reconstruct phase and polarization images with respect to diagnostically relevant representation systems and produce features useful for cancer detection. Moreover, our new approach decreases the reconstruction time to only few seconds from several hours when compared to the previously implemented technique [25], providing an important advance towards real-time image reconstruction.

In the past decades, there has been a significant interest in developing different imaging techniques that capture scattered light directly in the Fourier domain, such as light scattering angular spectroscopy [6], [28], [32], Fourier transform light scattering [27], angle-resolved low-coherence interferometry [38] and spatial-frequency domain imaging [31]. Some of these techniques have been successfully applied for detection of early cancer [51], providing further validation that Fourier coefficients indeed yield informative features. We remark that by our new approach, Fourier coefficients can be recovered without need to take the measurements in the Fourier domain, which is important because such measurements are not optimally tailored to the imaging guide such as fiber.

Finally, we mention that changing representation systems between image recovery and sampling has previously been applied to inverse problems arising in various image and signal processing applications (see [1], [23], and references therein). There, it is assumed that the imaging device of a known linear transformation provides image samples with respect to a specified sampling system, while the aim is to recover a representation of the image with respect to a different system chosen so that a good approximation of the image is obtained or the number of required samples is decreased. As in our paper, different representation systems are modeled by Riesz bases or frames of infinite-dimensional function-spaces. By contrast, the imaging device considered here produces pixel samples of a transformed image where the underlying transformation is unknown and is characterized through a calibration procedure.

## II. Reconstruction Framework

In this section we introduce our reconstruction framework. We start by presenting an infinite-dimensional imaging model in Section II-A. We then consider a simplified scalar-valued setting in Section II-B, where we derive a linear system and its regularized solution while providing flexibility in choosing different systems for calibration and image representation. We then extend our framework to vector-fields in Section II-C.

### A. Imaging Model and Reconstruction Problem

In imaging through fibers or other scattering media, an input optical vector-field $\mathbf {F}$ is related to its corresponding output $\tilde {\mathbf {F}}$ through an integral transformation with a spatially-varying kernel $\mathbf {G}$, also called Green’s function or point-spread function. Specifically, such transformation can be written as

\begin{equation*} \tilde {\mathbf {F}}(\mathbf {y}) = \int _{S} \mathbf {G}(\mathbf {y},\mathbf {x}) \mathbf {F}(\mathbf {x}) \,\mathrm {d}\mathbf {x},\tag{1}\end{equation*}

where $\mathbf {F}:S\rightarrow \mathbb {C}^{2}$ is a complex-valued vector-field representing the unknown optical field on the input plane $S\subseteq \mathbb {R}^{2}$, $\tilde {\mathbf {F}}:\mathbb {R}^{2}\rightarrow \mathbb {C}^{2}$ is a complex-valued vector-field on the output plane which can be sampled, and $\mathbf {G}:\mathbb {R}^{2}\times \mathbb {R}^{2}\rightarrow \mathbb {C}^{2\times 2}$ is some unknown bounded matrix-valued function [40]. In general, the kernel $\mathbf {G}$ is also time-dependent as it is affected by bending of the fiber and temperature. In this paper, we account for significant measurement noise but only consider imaging at a single time point; c.f. Section V. In particular, we consider the input field $\mathbf {F}$ to be an object with infinite resolution, and thus, we model $\mathbf {F}$ as an element of an infinite-dimensional function-space, such as the $\mathcal {L}^{2}$-space of square-integrable functions.

In our novel endoscopic imaging, we want to capture a full optical field (i.e. amplitude, phase and polarization) reflected from a human tissue inside the body, which is also called a wavefront, and which in this paper, we refer to as an image. In this terminology, $\mathbf {F}$ is an image observed indirectly at the input imaging plane $S$ at the end of the fiber inside the body, which is termed the *distal* facet of the fiber. The fiber then transports light from the distal facet to the *proximal* facet outside the body where the imaging sensor directly observes $\tilde {\mathbf {F}}$ at the output imaging plane. Then the question is how to recover the unknown $\mathbf {F}$ from the acquired samples of $\tilde {\mathbf {F}}$.

More concretely, given the pointwise measurements of the output vector-field $\tilde {\mathbf {F}}$ collected at the imaging sensor

\begin{equation*} \tilde {\mathbf {F}}(\mathbf {y}_{n}), \quad n=1,\ldots,N,\tag{2}\end{equation*}

where $\mathbf {y}_{n}\in \mathbb {R}^{2}$ and $N\in \mathbb {N}$ is the resolution of the imaging sensor, the goal is to recover the unknown function $\mathbf {F}$ via equation (1). It is important to note that these measurements will also contain noise introduced by the measurement procedure.

This linear inverse problem is especially challenging because both the spatially-varying kernel $\mathbf {G}$ as well as the eigenfunctions associated with the underlying integral transform (1) are unknown. Such eigenfunctions are termed modes of the fiber and their analytic form is available only for some limited fibers such as parabolic graded index multimode fibers [42].

To recover $\mathbf {F}$ from finitely many samples of $\tilde {\mathbf {F}}$ in scenarios where neither $\mathbf {G}$ nor the eigenfunctions are known, one strategy may be to employ a calibration procedure. Concretely, it is possible to design calibration input fields $\mathbf {E}_{m}$, $m=1,\ldots,M$, and to measure the corresponding output fields $\tilde {\mathbf {E}}_{m}$, which in line with the notation above are vector-valued functions related through the infinite-dimensional model given in (1). The advantage of calibration is that we now have access not only to the data given in (2) but also to the calibration data

\begin{equation*} \mathbf {E}_{m}, \quad \tilde {\mathbf {E}}_{m}(\mathbf {y}_{n}), ~ m=1,\ldots,M, ~ n=1,\ldots, N,\tag{3}\end{equation*}

which forms additional information with which to recover $\mathbf {F}$.

It is noted that while the output fields $\tilde {\mathbf {E}}_{m}$ are sampled at an output imaging sensor of resolution $N$, the calibration input fields $\mathbf {E}_{m}$ can be evaluated on a discretized grid whose resolution does not depend on any physical limitation imposed by the fiber or by the sensor collecting the transmitted image; it only depends on the resolution of the sensors used for calibration, which may be much larger than $M$. Therefore, as for the input $\mathbf {F}$, we model the inputs $\mathbf {E}_{m}$ as elements of an infinite-dimensional function-space. Thus, the representation of $\mathbf {F}$ as well as the device calibration can be considered with respect to a wide class of infinite-dimensional bases or over-complete systems that may not be orthogonal.

### B. Reconstruction of Scalar-Fields

We approach the general problem of recovering the complex vector-field $\mathbf {F}$ by first solving a simplified problem, which once solved will provide us with the methodology necessary to tackle the problem in its full generality in Section II-C. Specifically, we assume in this subsection that $F, \tilde {F}, E_{m}, \tilde {E}_{m}$ are scalar valued functions that take values in $\mathbb {C}$ rather than $\mathbb {C}^{2}$, and accordingly $G$ takes values in $\mathbb {C}$ rather than $\mathbb {C}^{2\times 2}$. We highlight this difference by using non-bold symbols.

We consider all fields on the input imaging plane $S$ as elements of the same function-space $\mathcal {F}$, such as the $\mathcal {L}^{2}$-space of square-integrable scalar-valued functions supported on $S$, with inner product defined as $\langle E,H \rangle:= \int _{S} E(\mathbf {x}) H^{*}(\mathbf {x}) \,\mathrm {d}\mathbf {x}$, for any $E,H\in \mathcal {F}$. We aim to recover $F\in \mathcal {F}$ at resolution $K\in \mathbb {N}$ in terms of some desired representation system $\{H_{k}\}_{k=1}^{K}$ in $\mathcal {F}$, using only the available data (2) and (3). Specifically, we aim to estimate the coefficients $\mathbf {f} = \begin{bmatrix} f_{1},\ldots, f_{K}\end{bmatrix}^{\top }\in \mathbb {C}^{K}$ of the $K$-term approximation of $F$ given as

\begin{equation*} F_{K}(\mathbf {x}):= \sum _{k=1}^{K} f_{k} H_{k}(\mathbf {x}), \quad \mathbf {x}\in S.\tag{4}\end{equation*}

Before turning to the computation of $f_{k}$ in (4), it is insightful to work through special cases of $S$ and $\{H_{k}\}_{k=1}^{K}$ that are particularly useful in practice. For instance, if we want to recover a Fourier representation of $F$ and $S:=[-1/2,1/2]^{2}\subseteq \mathbb {R}^{2}$, then $\{H_{k}\}_{k=1}^{K}$ is the $K$-dimensional Fourier basis $\{e^{2\pi \mathrm {i} \mathbf {k}\cdot \mathbf {x}}\}_{\mathbf {k}\in I_{K}}$ where $I_{K}:=\{\mathbf {k}=(k_{1},k_{2})\in \mathbb {Z}^{2}:k_{1},k_{2}=-\lceil \sqrt {K}/2\rceil,\ldots,\lceil \sqrt {K}/2\rceil -1\}$, $\mathbf {k}\cdot \mathbf {x}:=k_{1}x_{1}+k_{2}x_{2}$, $\mathbf {x}:=(x_{1},x_{2})\in S$, and (4) specializes to

\begin{equation*} F_{K}(\mathbf {x}):= \sum _{\mathbf {k}\in I_{K}} f_{\mathbf {k}} e^{2\pi \mathrm {i} \mathbf {k}\cdot \mathbf {x}}, \quad f_{\mathbf {k}}:=\int _{S} F(\mathbf {x})e^{-2\pi \mathrm {i} \mathbf {k}\cdot \mathbf {x}} \,\mathrm {d}\mathbf {x}.\tag{5}\end{equation*}

More generally, $\{H_{k}\}_{k=1}^{K}$ may contain the first $K$ elements of a Riesz basis in $\mathcal {F}$, such as B-spline wavelets for example, with its corresponding biorthogonal sequence denoted by $\{\breve {H}_{k}\}_{k=1}^{K}$, in which case (4) becomes $F_{K}(\mathbf {x}) = \sum _{k=1}^{K}\langle F,\breve {H}_{k} \rangle H_{k}$. Moreover, as we do not require an explicit form of the coefficients $f_{k}$, the notion of basis can be further relaxed to over-complete representation systems such as over-complete frames [15].

Returning to the key issue of approximating the coefficients $f_{k}$ in (4) from the given measurements (2)–(3), we write each $H_{k}$ in terms of the calibration functions $\left \{{E_{m}}\right \}_{m=1}^{M}$ as

\begin{equation*} H_{k}(\mathbf {x}) = \sum _{m=1}^{M} h_{m,k} E_{m}(\mathbf {x}) + \delta _{k}(\mathbf {x}), \quad \mathbf {x}\in S,\tag{6}\end{equation*}

for some coefficients $h_{m,k}\in \mathbb {C}$, whose computation we discuss below, and for some error term $\delta _{k}$. Since (1) is a linear transformation of $F$, by substituting $F$ with $F_{K}+(F-F_{K})$ in (1) and writing $F_{K}$ in terms of (4) and (6), we have

\begin{align*}&\hspace {-2.3pc}\tilde {F}(\cdot) = \sum _{k=1}^{K} \sum _{m=1}^{M} f_{k} h_{m,k} \tilde E_{m}(\cdot) + \sum _{k=1}^{K} f_{k} \int _{S} G(\cdot,\mathbf {x}) \\&\qquad \quad \times \,\delta _{k}(\mathbf {x}) \,\mathrm {d}\mathbf {x} +\int _{S} G(\cdot,\mathbf {x}) (F(\mathbf {x})-F_{K}(\mathbf {x})) \,\mathrm {d}\mathbf {x}.\tag{7}\end{align*}

By evaluating equation (7) at the measurement points $\{\mathbf {y}_{n}\}_{n=1}^{N}$, we obtain the following linear system

\begin{equation*} \mathbf {g} = \mathbf {E} \mathbf {H} \mathbf {f} + \boldsymbol {\varepsilon },\tag{8}\end{equation*}

where $\mathbf {g}:=[\tilde {F}(\mathbf {y}_{1}),\ldots, \tilde {F}(\mathbf {y}_{N})]^{\top }\in \mathbb {C}^{N}$, $\mathbf {E}\in \mathbb {C}^{N\times M}$ is the matrix with its $(n,m)$-th entry equal to $\tilde E_{m} (\mathbf {y}_{n})$, $\mathbf {H}\in \mathbb {C}^{M\times K}$ is the matrix with its $(m,k)$-th entry equal to $h_{m,k}$ and $\boldsymbol {\varepsilon }\in \mathbb {C}^{N}$ is an error term containing the last two terms in the right-hand-side of (7). In addition, the error term $\boldsymbol {\varepsilon }\in \mathbb {C}^{N}$ can be seen also as encapsulating measurement error incurred when measuring $\tilde {F}(\mathbf {y}_{n})$ and $\tilde {E}_{m}(\mathbf {y}_{n})$ in (2) and (3), respectively. We then opt to define the solution of (8) as

\begin{equation*} \bar {\mathbf {f}}:= \mathop {\mathrm {argmin}} _{\mathbf {f}\in \mathbb {C}^{K} } \,\left \{{ \| \mathbf {g} - \mathbf {E} \mathbf {H} \mathbf {f} \|_{2} + \lambda \mathcal {R}(\mathbf {f}) }\right \},\tag{9}\end{equation*}

where $\|\cdot \|_{2}$ denotes the Euclidean norm on $\mathbb {C}^{N}$, while the regularization term $\mathcal {R}$ and its parameter $\lambda \geq 0$ are described below. Once the coefficients $\bar {\mathbf {f}} = \begin{bmatrix}\bar f_{1},\ldots,\bar f_{K}\end{bmatrix}^{\top } \in \mathbb {C}^{K}$ are computed through (9), then in line with (4) we define the reconstruction of $F$ as the approximation given by

\begin{equation*} \bar F_{K}(\mathbf {x}):= \sum _{k=1}^{K} \bar f_{k} H_{k}(\mathbf {x}), \quad \mathbf {x}\in S.\tag{10}\end{equation*}

To obtain the explicit solution defined in (10), it remains to describe the procedure for computing the coefficients of matrix $\mathbf {H}$ and to define the regularization term $\mathcal {R}$.

First, observe that if the same system is used for calibration and reconstruction, then $\mathbf {H}=\mathbf {I}$. Otherwise, we can estimate $\mathbf {H}$ as follows. Using (6), we write $\langle H_{k}, E_{m^{\prime }} \rangle = \sum _{m=1}^{M} h_{m,k} \langle E_{m},E_{m^{\prime }} \rangle + \langle \delta _{k}, E_{m^{\prime }} \rangle $, $m^{\prime }=1,\ldots,M$, and thus, provided $\langle \delta _{k}, E_{m^{\prime }} \rangle \approx 0$, we can approximate $\mathbf {H}$ by

\begin{equation*} \begin{bmatrix} \langle E_{1},E_{1} \rangle & \ldots & \langle E_{M},E_{1} \rangle \\ \vdots & & \vdots \\ \langle E_{1},E_{M} \rangle & \ldots & \langle E_{M},E_{M} \rangle \end{bmatrix}^{-1} \begin{bmatrix} \langle H_{1},E_{1} \rangle & \ldots & \langle H_{K},E_{1} \rangle \\ \vdots & & \vdots \\ \langle H_{1},E_{M} \rangle & \ldots & \langle H_{K},E_{M} \rangle \end{bmatrix}\!.\end{equation*}

The first matrix above is known as the Gram matrix, which is equal to the identity if $\{E_{m}\}_{m=1}^{M}$ are orthonormal. We note that the accuracy of such estimation of matrix $\mathbf {H}$ and its condition number depend on the gap between the function-spaces spanned by $\{H_{k}\}_{k=1}^{K}$ and $\{E_{m}\}_{m=1}^{M}$ as well as on the conditioning of the Gram matrix. In general, it is required that $\{E_{m}\}_{m=1}^{M}$ form a good approximation for $\{H_{k}\}_{k=1}^{K}$.

Turning to the choice of the regularization term $\mathcal {R}$ in (9), in case it is absent, i.e. if $\lambda =0$, then the solution to (9) is equivalent to the least-squares solution $\bar {\mathbf {f}}:=((\mathbf {E} \mathbf {H})^{*} \mathbf {E} \mathbf {H})^{-1} (\mathbf {E} \mathbf {H})^{*} \mathbf {g}$. If the regularization term is given by $\mathcal {R}(\mathbf {f}):=\|\mathbf {f}\|_{2}$, then (9) is known as Tikhonov regularization and its solution is given by $\bar {\mathbf {f}}:=((\mathbf {E} \mathbf {H})^{*} \mathbf {E} \mathbf {H} + \lambda \mathbf {I})^{-1} (\mathbf {E} \mathbf {H})^{*} \mathbf {g}$. However, if $\mathbf {E} \mathbf {H}$ is badly conditioned, $\boldsymbol {\varepsilon }\neq 0$ and it is known a priori that only a few elements of $\{H_{k}\}_{k=1}^{K}$ are sufficient to represent $F$ well, then $\mathcal {R}(\mathbf {f}):=\|\mathbf {f}\|_{0}$ is an appropriate choice of the regularization term. This is known as the $\ell _{0}$-regularization, where the $\ell _{0}$-norm $\|\mathbf {f}\|_{0}$ is defined as the number of non-zero entries in $\mathbf {f}$. The $\ell _{0}$-regularization bypasses the ill-conditioning by imposing sparsity in the solution $\bar F_{K}$ with respect to $\{H_{k}\}_{k=1}^{K}$. In practice, solving the minimization problem with such a non-convex $\ell _{0}$-term is computationally difficult, so typically an $\ell _{1}$-relaxation is considered instead. The corresponding relaxed minimization problem can then be solved by fast iterative algorithms [10], [11]. In addition, the parameter $\lambda $, which controls the strength of the regularization, can be chosen by cross-validation techniques [20].

We conclude this subsection with a discussion on the accuracy and robustness of the solution defined in (10).

The reconstruction error can be quantified by the magnitude of $F-\bar {F}_{K}=(F-F_{K})+(F_{K}-\bar {F}_{K})$. The magnitude of $F-F_{K}$ depends of how well $F$ can be represented by its $K$-term approximation with respect to $\{H_{k}\}_{k=1}^{K}$, and thus it is expected to decrease with increasing $K$. On the other hand, the magnitude of $F_{K}-\bar {F}_{K}$ depends on the conditioning of $\mathbf {E}\mathbf {H}$ and the error term $\boldsymbol {\varepsilon }$ in (8), and thus, it is expected to increase with increasing $K$ and $M,N$ fixed. If the resolution $K$ at which we reconstruct is increased, we also need to increase $M$ and $N$. However, it may be possible to attain higher resolutions if some form of regularization is used when solving (8).

As previously noted, the error term $\boldsymbol {\varepsilon }$ contains the measurement error as well as the last two terms in (7), which can be disregarded provided $F - F_{K}$ and $\delta _{k}$ are small or they lie in the span of the eigenfunctions corresponding to a small singular value. Thus, small $\boldsymbol {\varepsilon }$ requires that $\{H_{k}\}_{k=1}^{K}$ and $\{E_{m}\}_{m=1}^{M}$ form a good approximation for $F$ or for the eigenfunctions with large singular values. However, if the singular values of the underlying integral operator accumulate at zero, the conditioning of the matrix $\mathbf {E}$ may become worse if the span of $\{E_{m}\}_{m=1}^{M}$ includes too many eigenfunctions including those corresponding to a small singular value. Loosely speaking, $\{E_{m}\}_{m=1}^{M}$ should form a good representation for the span of the eigenfunctions, excluding those corresponding to small singular values if they exist. However, as we do not have access to the true eigenfunctions, we do not have control over the ill-conditioning introduced by using a particular choice of $\{E_{m}\}_{m=1}^{M}$. Thus, the use of regularization in solving (8) becomes crucial in order to obtain a robust solution.

### C. Reconstruction of Vector-Fields

We now extend the scalar-field reconstruction framework developed in Section II-B to the more general vector-field problem presented in Section II-A. To begin with, let $\mathbf {F}:= \begin{bmatrix} F^{h},\,\, F^{v} \end{bmatrix}^{\top } \mathop{\mapsto }\limits^{ (1)} \tilde {\mathbf {F}}:= \begin{bmatrix} \tilde {F}^{h},\,\, \tilde {F}^{v} \end{bmatrix}^{\top }$ be the complex-vector-valued functions related as in equation (1), where the superscripts $h$ and $v$ correspond to the horizontal and vertical polarizations of the optical field, respectively. The goal is to recover both polarizations $F^{h}$ and $F^{v}$, which are scalar-valued functions, by using data (2)–(3). Since each $\tilde {F}^{h}$ and $\tilde {F}^{v}$ depends on both $F^{h}$ and $F^{v}$, instead of reconstructing each polarization independently, we consider their joint reconstruction. As we will see in Section IV-A, the reconstruction of each individual polarization can be improved if they are reconstructed jointly. Nevertheless, as it will be demonstrated below, we can still use the framework introduced in Section II-B, as long as the calibration inputs can form a representation system for vector-valued functions such as $\mathbf {F}$. To ensure this is the case, rather than straightforwardly sampling the vector-valued calibration inputs in (3), we sample the following two related forms

\begin{align*} \mathbf {A}_{m}:=&\begin{bmatrix} E_{m}^{h} \\ E_{m}^{v} \end{bmatrix} \overset { (1)}{\mapsto } \tilde {\mathbf {A}}_{m}:= \begin{bmatrix} \tilde {A}_{m}^{h} \\ \tilde {A}_{m}^{v} \end{bmatrix}\!, \\ \mathbf {B}_{m}:=&\begin{bmatrix} E_{m}^{h} \\ b E_{m}^{v} \end{bmatrix} \overset { (1)}{\mapsto } \tilde {\mathbf {B}}_{m}:= \begin{bmatrix} \tilde {B}_{m}^{h} \\ \tilde {B}_{m}^{v} \end{bmatrix}\!,\tag{11}\end{align*}

where $m=1,\ldots,M$, $b:= \mathrm {e}^{\beta \mathrm {i} }$ for a fixed $\beta \in (0,2\pi)$ and $E_{m}^{h},E_{m}^{v},\tilde {A}_{m}^{h},\tilde {A}_{m}^{v},\tilde {B}_{m}^{h},\tilde {B}_{m}^{v}$ are some scalar-valued functions. To make clear the motivation to sample according to (11), observe that if $\{E_{m}^{h}\}_{m=1}^{M}$ and $\{ E_{m}^{v}\}_{m=1}^{M}$ are representation systems for $F^{h}$ and $F^{v}$ respectively, then $\{\mathbf {A}_{m} - \mathbf {B}_{m}\}_{m=1}^{M}$ and $\{\mathbf {A}_{m} - b^{*} \mathbf {B}_{m}\}_{m=1}^{M}$ are representation systems for $\begin{bmatrix} 0, ~ F^{v} \end{bmatrix}^{\top }$ and $\begin{bmatrix} F^{h}, ~0 \end{bmatrix}^{\top }$, respectively. In other words,

\begin{equation*} \bigl \{\mathbf {A}_{m}-\mathbf {B}_{m}, \mathbf {A}_{m}-b^{*}\mathbf {B}_{m}: m=1,\ldots,M\bigr \}\tag{12}\end{equation*}

can be used to represent the complex vector-valued $\mathbf {F}$.

Mimicking the reasoning of the previous subsection, we proceed by approximating $F^{h}$ and $F^{v}$ with respect to some desired representation systems $\{H_{k}^{h}\}_{k=1}^{K}$ and $\{H_{k}^{v}\}_{k=1}^{K}$. Namely, we aim to recover $F^{h}_{K}(\mathbf {x}):=\sum _{k=1}^{K} f_{k}^{h} H_{k}^{h}(\mathbf {x})$ and $F^{v}_{K}(\mathbf {x}):=\sum _{k=1}^{K} f_{k}^{v} H_{k}^{v}(\mathbf {x})$, where we first write these representations in terms of the calibration inputs, i.e. $H^{h}_{k}(\mathbf {x})=\sum _{m=1}^{M} h_{m,k}^{h} E_{m}^{h}(\mathbf {x})+\delta ^{h}_{k}(\mathbf {x})$ and $H^{v}_{k}(\mathbf {x})=\sum _{m=1}^{M} h_{m,k}^{v} E_{m}^{v}(\mathbf {x})+\delta ^{v}_{k}(\mathbf {x})$, for some coefficients $f_{k}^{h},f_{k}^{v},h_{m}^{h},h_{m}^{v}\in \mathbb {C}$ and error terms $\delta ^{h}_{k},\delta ^{v}_{k}$. It follows that

\begin{align*} \mathbf {F}(\mathbf {x})=&\sum _{k=1}^{K} \sum _{m=1}^{M} f_{k}^{h} h_{m,k}^{h} \begin{bmatrix} E_{m}^{h}(\mathbf {x}) \\ 0 \end{bmatrix} \\&+\, \sum _{k=1}^{K} \sum _{m=1}^{M} f_{k}^{v} h_{m,k}^{v} \begin{bmatrix} 0 \\ E_{m}^{v}(\mathbf {x}) \end{bmatrix} \\&+\, \sum _{k=1}^{K} \begin{bmatrix} f_{k}^{h}\delta _{k}^{h}(\mathbf {x}) \\ f_{k}^{v}\delta _{k}^{v}(\mathbf {x}) \end{bmatrix} + \begin{bmatrix} F^{h}(\mathbf {x})-F^{h}_{K}(\mathbf {x})\\ F^{v}(\mathbf {x})-F^{v}_{K}(\mathbf {x}) \end{bmatrix}\!.\tag{13}\end{align*}

Since $\begin{bmatrix} E_{m}^{h}(\mathbf {x}), 0 \end{bmatrix}^{\top } = (1-b^{*})^{-1} \left ({\mathbf {A}_{m}(\mathbf {x}) - b^{*} \mathbf {B}_{m}(\mathbf {x}) }\right)$ and $\begin{bmatrix} 0, E_{m}^{v}(\mathbf {x}) \end{bmatrix}^{\top } = (1-b)^{-1} \left ({\mathbf {A}_{m}(\mathbf {x}) - \mathbf {B}_{m} (\mathbf {x}) }\right)$, we obtain

\begin{align*}&\hspace {-0.5pc}\tilde {\mathbf {F}}(\cdot) \approx \frac {1}{1-b^{*}} \sum _{k=1}^{K} \sum _{m=1}^{M} f_{k}^{h} h_{m,k}^{h} \left ({\tilde {\mathbf {A}}_{m}(\cdot) - b^{*} \tilde {\mathbf {B}}_{m} (\cdot) }\right) \\&\qquad \quad \qquad \qquad + \,\frac {1}{1-b} \sum _{k=1}^{K} \sum _{m=1}^{M} f_{k}^{v} h_{m,k}^{v} \left ({\tilde {\mathbf {A}}_{m}(\cdot) - \tilde {\mathbf {B}}_{m} (\cdot) }\right)\!,\end{align*}

by applying (1) to (13), provided the two last terms in (13) are small or they become small after applying (1). By using the pointwise measurements from (2) and (11), this leads to

\begin{equation*} \mathbf {g} = \mathbf {E} \mathbf {H} \mathbf {f} + \boldsymbol {\varepsilon }, \quad \mathbf {E}:= \begin{bmatrix} a^{*}\left ({\mathbf {A} - b^{*}\mathbf {B}}\right) & a\left ({\mathbf {A} - \mathbf {B}}\right) \end{bmatrix}\!,\tag{14}\end{equation*}

where $\mathbf {g}:=\begin{bmatrix} \tilde F^{h}(\mathbf {y}_{1}), \tilde F^{v}(\mathbf {y}_{1}), \cdots, \tilde F^{h}(\mathbf {y}_{N}), \tilde F^{v}(\mathbf {y}_{N}) \end{bmatrix}^{\top }\in \mathbb {C}^{2N}$, $\mathbf {f}:= \begin{bmatrix} f^{h}_{1}, \cdots, f^{h}_{K}, f^{v}_{1}, \cdots, f^{v}_{K} \end{bmatrix}^{\top }\in \mathbb {C}^{2K}$, $\boldsymbol {\varepsilon }\in \mathbb {C}^{2N}$ is the error term, $a:=1/(1-b)\in \mathbb {C}$, $\mathbf {A},\mathbf {B}\in \mathbb {C}^{2N\times M}$ are defined as

\begin{align*} \mathbf {A}:=&\begin{bmatrix} \tilde {A}_{1}^{h}(\mathbf {y}_{1}) & \ldots & \tilde {A}_{M}^{h}(\mathbf {y}_{1}) \\ \tilde {A}_{1}^{v}(\mathbf {y}_{1}) & \ldots & \tilde {A}_{M}^{v}(\mathbf {y}_{1}) \\ & \ddots & \\ \tilde {A}_{1}^{h}(\mathbf {y}_{N}) & \ldots & \tilde {A}_{M}^{h}(\mathbf {y}_{N}) \\ \tilde {A}_{1}^{v}(\mathbf {y}_{N}) & \ldots & \tilde {A}_{M}^{v}(\mathbf {y}_{N}) \\ \end{bmatrix}\!\!, \\ \mathbf {B}:=&\begin{bmatrix} \tilde {B}_{1}^{h}(\mathbf {y}_{1}) & \ldots & \tilde {B}_{M}^{h}(\mathbf {y}_{1}) \\ \tilde {B}_{1}^{v}(\mathbf {y}_{1}) & \ldots & \tilde {B}_{M}^{v}(\mathbf {y}_{1}) \\ & \ddots & \\ \tilde {B}_{1}^{h}(\mathbf {y}_{N}) & \ldots & \tilde {B}_{M}^{h}(\mathbf {y}_{N}) \\ \tilde {B}_{1}^{v}(\mathbf {y}_{N}) & \ldots & \tilde {B}_{M}^{v}(\mathbf {y}_{N}) \\ \end{bmatrix}\!\!,\end{align*}

and $\mathbf {H}\in \mathbb {C}^{2M\times 2K}$ is a block-diagonal matrix $\mathrm {diag}(\mathbf {H}^{h},\mathbf {H}^{v})$, where $\mathbf {H}^{h}\in \mathbb {C}^{M\times K}$ is such that its $(m,k)$-th entry is $h^{h}_{m,k}$ and $\mathbf {H}^{v}\in \mathbb {C}^{M\times K}$ is such that its $(m,k)$-th entry is $h^{v}_{m,k}$. We propose to solve the linear system (14) in a similar manner to that used in (9). Finally, once (14) is solved for the coefficients $\bar {\mathbf {f}}= \begin{bmatrix} \bar f^{h}_{1}, \ldots, \bar f^{h}_{K}, \bar f^{v}_{1}, \ldots, \bar f^{v}_{K} \end{bmatrix}^{\top }\in \mathbb {C}^{2K}$, we can define the reconstructions of $F^{h}$ and $F^{v}$ as $\bar F_{K}^{h}(\cdot):=\sum _{m=1}^{K} \bar f_{k}^{h} H_{k}^{h}(\cdot)$ and $\bar F_{K}^{v}(\cdot):=\sum _{m=1}^{K} \bar f_{k}^{v} H_{k}^{v}(\cdot)$. Observe that using $\ell _{1}$-regularization in this case imposes sparsity in the reconstructions $\bar F_{K}^{h}$ and $\bar F_{K}^{v}$ with respect to $\{H_{k}^{h}\}_{k=1}^{K}$ and $\{ H_{k}^{v}\}_{k=1}^{K}$, respectively. Also similarly as before, we note that matrices $\mathbf {H}^{h}$ and $\mathbf {H}^{v}$ are identities when the reconstruction functions $H_{k}^{h}, H_{k}^{v}$ and the calibration functions $E_{m}^{h}, E_{m}^{v}$ are the same, otherwise they can be computed approximately from the equations $\langle H_{k}^{h}, E_{m^{\prime }}^{h} \rangle \approx \sum _{m=1}^{M} h^{h}_{m,k} \langle E_{m}^{h}, E_{m^{\prime }}^{h} \rangle $ and $\langle H_{k}^{v}, E_{m^{\prime }}^{v} \rangle \approx \sum _{m=1}^{M} h^{v}_{m,k} \langle E_{m}^{v}, E_{m^{\prime }}^{v} \rangle $, $m^{\prime }=1,\ldots,M$.

Finally, we note that in order to reduce the impact of noise it may be possible to include measurements of additional phase-shifts of the calibration functions. In addition to the calibration inputs $\mathbf {A}_{m}$ and $\mathbf {B}_{m}$ in (11), it may also be possible to measure $\mathbf {C}_{m}:= \begin{bmatrix} E_{m}^{h}, c E_{m}^{v} \end{bmatrix}^{\top }\mathop{\mapsto }\limits^{ (1)} \tilde {\mathbf {C}}_{m}:= \begin{bmatrix} \tilde {C}_{m}^{h}, \tilde {C}_{m}^{v} \end{bmatrix}^{\top }$, where $c$ is such that $c\neq b$ and $b+c\neq 2$. Thus, rather than using (12), we can use $\left\{{ a^{*} \left({\mathbf {A}_{m} - \tfrac {b^{*}}{2} \mathbf {B}_{m} - \tfrac {c^{*}}{2} \mathbf {C}_{m} }\right), \,\,a \left({\mathbf {A}_{m} - \tfrac {1}{2} \mathbf {B}_{m} - \tfrac {1}{2} \mathbf {C}_{m} }\right):\,\,m=1,\ldots,M }\right\}$, where $a:= 1/\left ({1-b/2 - c/2 }\right)$ is finite given that $b+c\neq 2$. We then proceed as above but in place of (14) get

\begin{align*} \mathbf {g}=&\mathbf {E} \mathbf {H} \mathbf {f} + \boldsymbol {\varepsilon }, \\ \mathbf {E}:=&\begin{bmatrix} a^{*}\left ({\mathbf {A} - \frac {b^{*}}{2} \mathbf {B} - \frac {c^{*}}{2} \mathbf {C}}\right) \quad a\left ({\mathbf {A} - \frac {1}{2} \mathbf {B} - \frac {1}{2} \mathbf {C}}\right) \end{bmatrix}\!,\tag{15}\end{align*}

where $\mathbf {E}$ now includes $\mathbf {C}\in \mathbb {C}^{2N\times M}$ containing the outputs $\tilde {\mathbf {C}}_{m}$. As we will see in Section IV, augmenting the calibration data in such a way is indeed an effective manner to decrease the influence of the measurement noise on the reconstruction.

## III. Fourier Coefficients as Informative Features

Since inhomogeneities on a cellular scale caused by cancer result in increased scattering of an optical field reflected from a tumouros tissue [21], it is expected that they also result in higher spatial frequencies of the corresponding optical field. Hence, we propose that by representing such optical fields in a Fourier basis and by inspecting the associated Fourier coefficients it is possible to detect the increased scattering and thereby gain insight into the disease status of the tissue.

In this section, we focus on the merits of the Fourier coefficients as indicative features of increased phase scattering. By using simulated data, we show how increased changes in phase result in a slower decay of the corresponding Fourier coefficients and how this effect can be quantified. In the next section, we confirm these findings on real biological data, where we use the framework developed in Section II to recover tissue images directly in a Fourier basis and demonstrate that the Fourier coefficients are indeed useful for detecting cancer.

### A. Fourier Coefficients of a One-Dimensional Example

**Fig. 1.**
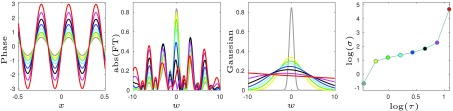
Higher phase oscillations (larger $\tau $) results in a slower decay of the Fourier coefficients (larger $\sigma $).

We first consider a simple 1D example to illustrate the effect of increased phase oscillations on the decay of the corresponding Fourier coefficients. We compute the Fourier coefficients of eight different functions $F^{(j)}(x)=R(x)\exp ({ \mathrm {i} P^{(j)}(x)})$, $j=1,\ldots,8$, with the same amplitude $R$ but different phase $P^{(j)}$ defined on the interval $x\in I:=[-1/2,1/2]$. For illustration purposes we take $R(x):=\exp (-x^{2})$ and $P^{(j)}(x):=\tau ^{(j)}\sin (20x)$, where $0< \tau ^{(1) }< \cdots < \tau ^{(8) } < 2\pi $, so that different phase functions exhibit different degrees of oscillations. These phase functions are shown in the first panel of Fig. 1. Since the Fourier basis on $I$ is given by $\{e^{2\pi \mathrm {i} k x}\}_{k\in \mathbb {Z}}$, for each $F^{(j)}$ we compute its first 20 Fourier coefficients as

\begin{equation*} f^{(j)}_{k}:= \int _{I} F^{(j)}(x)e^{-2\pi \mathrm {i} kx} \,\mathrm {d} x,\end{equation*}

where $k=-10,\ldots,9$, and approximate its Fourier transform by the classical Whittaker–Shannon interpolation formula $\sum _{k}f^{(j)}_{k}\mathrm {sinc}(w-k)$, $w\in \mathbb {R}$. The absolute value of the approximated Fourier transform for each $j$ is shown in the second panel of Fig. 1. Finally, we quantify the decay of the Fourier coefficients by the standard deviation $\sigma ^{(j)}$ of a Gaussian function $a^{(j)}\exp (-(w-c^{(j)})^{2}/(2(\sigma ^{(j)})^{2}))$ fitted to the amplitude of the approximated Fourier transform on interval $w\in [-10,10$). The fitted Gaussian functions are shown in the third panel of Fig. 1. From the fourth panel of Fig. 1, we observe that an increased magnitude of the phase oscillations $\tau ^{(j)}$ results in an increased standard deviation $\sigma ^{(j)}$. It is important to note that although in this example the zeros of the different phase functions coincide, the same effect is observed even if this is not the case. Also, if the frequency of the phase oscillation is increased while their magnitude is kept constant, then $\sigma ^{(j)}$ would increase as well.

The takeaway message from this simple example is that representing a signal with respect to a Fourier basis is especially useful to identify variations in oscillating phase, and that the decay of the corresponding Fourier coefficients is sensitive to phase scattering in a manner that can be easily identified. As we will see in the remainder of the paper, these observations remain true also in higher dimensional practical examples.

### B. Fourier Coefficients of Simulated Tissue Images

We now generalize our observations to 2D functions. For this purpose, we create a model mimicking tissue samples with a different level of phase oscillations, which we then use to generate images and compute their Fourier coefficients.

In our model, we use randomness to achieve certain variability across different samples and two different parameters to control the degree of phase oscillations. In particular, our model corresponds to a function $F(\mathbf {x}):=R(\mathbf {x})\exp (\mathrm {i} P(\mathbf {x}))$, $\mathbf {x}\in S$, where the original space-domain $S:=[-1/2,1/2]^{2}$ is discretized into a $700\times 700$ grid, while $R$ and $P$ are chosen randomly as we now describe. The phase function $P:=P^{(\tau,\rho)}$ depends on two given parameters $\tau $ and $\rho $, controlling the amplitude and the frequency of phase oscillations, respectively. To produce $P$, first $800\times 800$ pixel-values are chosen uniformly at random from [−1, 1], which are then filtered by using MATLAB’s function ‘imgaussfilt’ with the smoothing parameter $\rho $. Following this step, only $700\times 700$ pixels are kept by removing 50 pixels from each boundary and such image is then rescaled so that all phase values are between $[-\tau,\tau]$, $\tau \in [0,\pi]$. The amplitude $R$ is selected as the sum of $\exp (-50\|\mathbf {x}\|_{2}^{2})/1000$ and five additional Gaussian functions $\exp (-\|\mathbf {x}-\mathbf {c}\|_{2}^{2}/d)/2000$ with randomly chosen $\mathbf {c}$ and $d$.

**Fig. 2.**
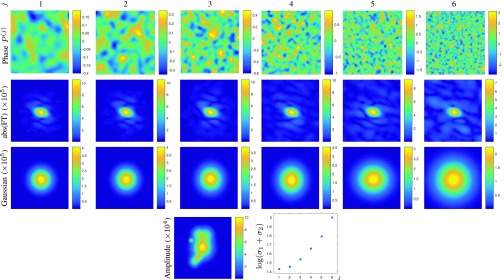
Six simulated images with the same amplitude but different phase, which are generated from our model with increasing $\tau ^{({j}{)}}/\rho ^{(j{)}}$, ${j}=\textsf {1},\ldots,\textsf {6}$, so that larger $\tau ^{({j}{)}}/\rho ^{({j}{)}}$ characterizes larger phase oscillations. In the scatter plot, we report the logarithm of the sum of parameters $\sigma _{\textsf {1}}^{({j}{)}}$ and $\sigma _{\textsf {2}}^{({j}{)}}$ of the Gaussian fitted to the amplitude of the Fourier transform abs(FT), revealing that increased $\tau ^{({j}{)}}/\rho ^{({j}{)}}$ correlates with larger $\sigma _{\textsf {1}}^{({j}{)}}+\sigma _{\textsf {2}}^{({j}{)}}$.

In Fig. 2 we demonstrate how changing phase parameters $\tau $ and $\rho $ while keeping amplitude fixed changes the decay of Fourier coefficients. Specifically, we use six values $(\rho ^{(j)},\tau ^{(j)})$, $j=1,\ldots,6$ to create six functions $F^{(j)}$, where $0< \tau ^{(1) }< \cdots < \tau ^{(6) }\leq \pi $ and $0.025< (\rho ^{(1) })^{-1}< \cdots < (\rho ^{(6) })^{-1}\leq 0.125$ are increasing logarithmically. Similarly to the 1D example of Fig. 1, the decay of corresponding Fourier coefficients is measured by standard deviation of a Gaussian function $a\exp (-(x_{1}-c_{1})^{2}/(2\sigma _{1}^{2})-(x_{2}-c_{2})^{2}/(2\sigma _{2}^{2}))$ fitted to the absolute value of the Fourier transform approximated from the first $20\times 20$ Fourier coefficients, which are computed using the formula in (5). In Fig. 2, for each $F^{(j)}$ we report the sum of the standard deviations $\sigma _{1}^{(j)}+\sigma _{2}^{(j)}$ of the fitted Gaussian, thereby observing that increased phase oscillations, i.e. increased $\tau ^{(j)}/\rho ^{(j)}$, results in slower decay of the corresponding Fourier coefficients, i.e. larger $\sigma _{1}^{(j)}+\sigma _{2}^{(j)}$.

**Fig. 3.**
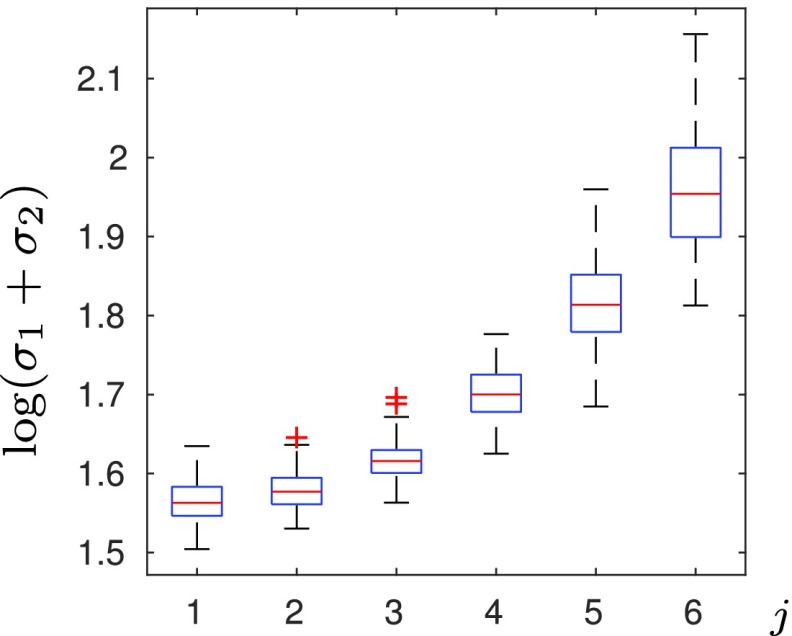
For each of the six categories, we generate 100 images with different phase and amplitude from our tissue model with fixed parameters $\tau ^{(\textsf {j}{)}}$ and $\rho ^{({j}{)}}$, ${j}=\textsf {1},\ldots,\textsf {6}$, and compute corresponding $\sigma _{\textsf {1}}^{({j}{)}}+\sigma _{\textsf {2}}^{({j}{)}}$.

Next, in Fig. 3, for each $(\rho ^{(j)},\tau ^{(j)})$, $j=1,\ldots,6$, chosen as in Fig. 2, we generate 100 images using our model (with each image having a different phase and a different amplitude) and we report the value $\sigma _{1}^{(j)}+\sigma _{2}^{(j)}$ of the fitted Gaussian. We observe the same trend in the decay of the Fourier coefficients in Fig. 3 as in Fig. 2, but now across 600 different images.

We note that the features extracted from Fourier coefficients as we described above have three additional useful properties. First, since the amplitude of the Fourier transform is invariant to the shifts of the corresponding complex function in its space-domain, the features that we extract are invariant to the shifts of the tissue images in their space-domain. Second, the quality of the recovered phase in the space-domain is dependent on a phase unwrapping procedure and is thus highly sensitive to noise, which means that phase may bear more information in the Fourier-domain than in the space-domain. Third, once the Fourier coefficients are recovered, each image can easily be represented in both the Fourier and the space-domain, allowing for additional flexibility.

## IV. Experimental Results

Having established the utility of Fourier coefficients in quantifying phase scattering in Section III, we now apply the reconstruction framework developed in Section II to measurements obtained experimentally by a fiber endoscope, which is described in the supplementary material available online in the supplementary file/multimedia tab. In Section IV-A, we first demonstrate the recovery of a synthetic holographic image with a known ground-truth that can be used for validation. Next, in Section IV-B, we apply our method to biological images of tissue samples taken from mice and demonstrate that reconstruction with respect to a Fourier basis can be used as a diagnostic indicator of early tumorigenesis.

### A. Reconstruction of a Synthetic Holographic Image

**Fig. 4.**
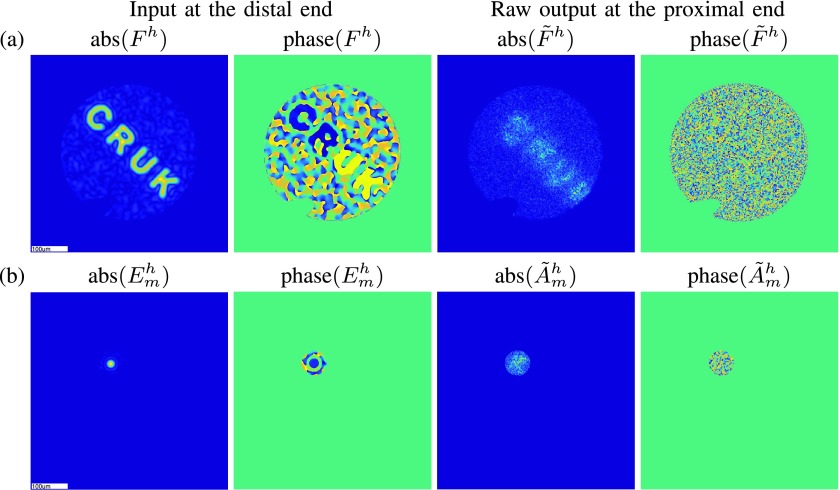
(a): Amplitude and phase of the horizontal polarization of the ground-truth synthetic holographic image at the distal end and the corresponding raw output at the proximal end. Due to space limitation, the vertical polarization is presented as Fig. 13 in the supplementary material. (b): Amplitude and phase of the horizontal polarization of one calibration input ($\mathbf {\textsf {A}}_{m}$ of Eq. (11)) at the distal end and at the proximal end. More information about the calibration inputs can be found in the supplementary material, Section I-B.

To demonstrate our reconstruction algorithm on experimental data we first reconstruct a synthetic holographic image, which was generated experimentally as explained in the supplementary material, Section I-C. Since in this case we have access to the ground-truth image, we can visually assess the quality of our proposed imaging methodology. Specifically, using the raw output of the holographic image shown in Fig. 4(a), we test our general reconstruction framework in combination with different representation systems as well as different regularization terms.

The MCF system is calibrated using input and output pairs as exemplified in Fig. 4(b). Given the localized confinement of light in MCF, for efficient calibration, several input and output calibration functions are evaluated in parallel. However, for the reconstruction, individual calibration functions are separated from the rest by evaluating each of them only over a circular region around the center of the corresponding Gaussian-like spot. In particular, the calibration inputs in Fig. 4(b) are evaluated on a $1200\times 1200$ grid and translated to $M=936$ different locations across the input imaging plane. Each output is evaluated at $N=34973$ pixels at the output imaging plane. Thus, considering the two polarization states, the dimension of the system matrix in (15) is $1872\times 69946$.

**Fig. 5.**
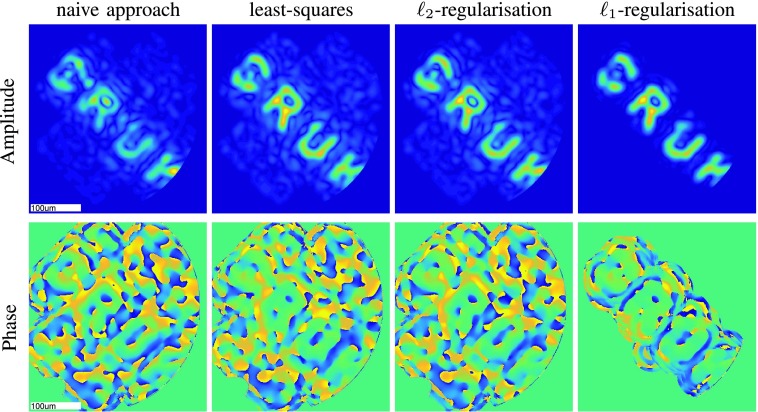
Reconstructed amplitude and phase (horizontal polarization) of the holographic image from Fig. 4(a) with respect to the calibration functions such as those in Fig. 4(b), using naive, least-squares, $\ell _{\textsf {2}}$ and $\ell _{\textsf {1}}$ approaches. The regularization parameter in the $\ell _{\textsf {2}}$ and $\ell _{\textsf {1}}$-regularization is $\lambda =\textsf {0.3}$ and $\lambda =\textsf {0.267}$, respectively. The vertical polarization is presented in the supplementary material, Fig. 14. The reconstruction time^1^ is under 1s for the naive inversion and the $\ell _{\textsf {1}}$-regularization, while it is around 4s for the least-squares and the $\ell _{\textsf {2}}$-regularization.

In Fig. 5, we recover the amplitude and the phase of the horizontal and vertical polarizations of the holographic image from raw endoscopic measurements using different inversion techniques while reconstructing with respect to the calibration coefficients. In particular, we solve (15) where $\mathbf {H}^{h}=\mathbf {H}^{v}=\mathbf {I}$, by inverting the linear system in four different ways:

1. the naive inversion $\bar {\mathbf {f}}:= \mathbf {E}^{*} \mathbf {g} $, which corresponds to the principle of phase conjugation as it assumes $\mathbf {E}^{*}\mathbf {E}=\mathbf {I}$,
2. the least-squares approach $\bar {\mathbf {f}}:=(\mathbf {E}^{*} \mathbf {E})^{-1} \mathbf {E}^{*} \mathbf {g}$,
3. the $\ell _{2}$-regularization $\bar {\mathbf {f}}:= (\mathbf {E}^{*} \mathbf {E} + \lambda \mathbf {I})^{-1} \mathbf {E}^{*} \mathbf {g}$, and,
4. the $\ell _{1}$-regularization $\bar {\mathbf {f}}:= \mathop {\mathrm {argmin}} _{\mathbf {f}\in \mathbb {C}^{2M} } \| \mathbf {g} - \mathbf {E} \mathbf {f} \|_{2}+\lambda \|\mathbf {f}\|_{1}$, using the iterative solver [12].

Fig. 5 shows that $\ell _{1}$-regularization performs well when compared to the other approaches. In fact, since this image is sparse with respect to the calibration inputs, $\ell _{1}$ successfully removes significant noise while preserving the image details.^1^The reconstruction time is time needed to solve the corresponding linear system and produce amplitude and phase images for two polarizations and is computed as an average over 10 runs using Matlab on Intel(R) Core(TM) i5-4670 CPU @ 3.40GHz [4 CPUs] 3401 Mhz.

**Fig. 6.**
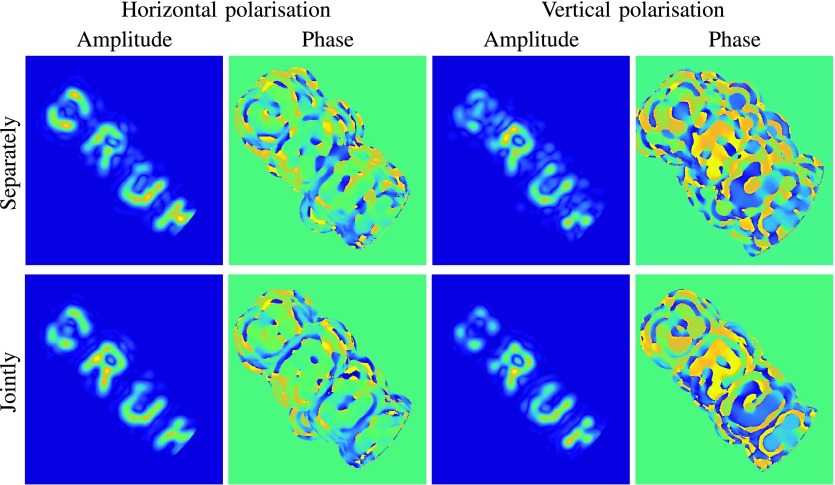
Top row: each polarization is reconstructed separately by solving a variant of Eq. (15), where only one polarization is considered at the time. Bottom row: the two polarizations are reconstructed jointly at the same time via (15). All linear systems are solved via $\ell _{\textsf {1}}$-regularization, while the corresponding reconstructions via least squares are shown in the supplementary material, Fig. 15. The scale bar is the same as in Fig. 5.

Next, we compare the proposed approach to its naive version, which reconstructs each polarization separately and thereby excludes the interaction between different polarizations. Specifically, in the upper panels of Fig. 6, the horizontal polarization $F^{h}$ is reconstructed by solving a variant of Eq. (15) for the unknown vector $[f^{h}_{1},\ldots,f^{h}_{K}]^{\top }$, where only the samples of the horizontal polarization $\tilde {F}^{h}$ are considered and the corresponding calibration measurements are $E^{h}_{m} \mapsto a^{*} \left({\tilde {A}_{m} - \tfrac {b^{*}}{2} \tilde {B}_{m} - \tfrac {c^{*}}{2} \tilde {C}_{m}}\right)$. Similarly, the vertical polarization $F^{v}$ is reconstructed by solving another linear system, which accounts for the vertical polarization only. By comparing these reconstructions to those in the lower panels of Fig. 6, we see that by reconstructing different polarizations jointly via (15), we improve the reconstruction of each individual polarization.

**Fig. 7.**
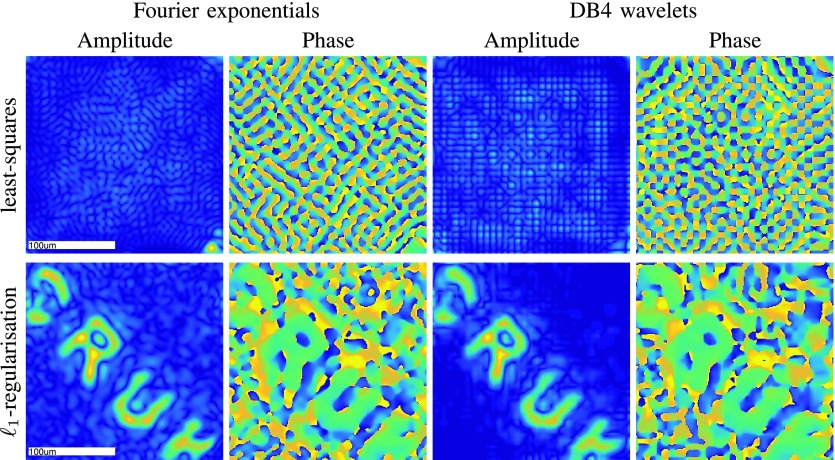
Reconstructed images (horizontal polarization) with respect to the different bases using two inversion approaches. We used $\textsf {32}\times \textsf {32}$ Fourier exponentials/DB4 wavelets. In the $\ell _{\textsf {1}}$-regularization $\lambda =\textsf {0.25}$ and the reconstruction time^1^ is around 30s.

Finally, in Fig. 7, we reconstruct the holographic image with respect to different representation systems, namely we solve (15) where both $\mathbf {H}^{h}$ and $\mathbf {H}^{v}$ correspond to a Fourier or a wavelet basis with cardinality $K=1024$. Specifically, we choose both $\{H_{k}^{h}\}_{k=1}^{K}$ and $\{H_{k}^{v}\}_{k=1}^{K}$ to be

(i) in the Fourier case, $\{\exp (2\pi \mathrm {i} (k_{1}\,\,x_{1} + k_{2}\,\,x_{2})): k_{1},k_{2}=-\sqrt {K}/2,\ldots,\sqrt {K}/2-1\}$, $\mathbf {x}=(x_{1},x_{2})\in [-1/2,1/2]^{2}$,
(ii) in the wavelet case, tensor-products of $\sqrt {K}~1\text{D}$ boundary-corrected Daubechies wavelets with four vanishing moments (DB4) from [19].

Fig. 7 shows that least-squares fails to give a useful estimate, conveying that it is crucial to use regularization. Although least-squares could still be used when $K\ll M$, small $K$ does not necessarily lead to a good approximation of the image, and so to achieve the desired resolution one would need to increase the number of calibration measurements $M$, which is undesirable as it would incur additional experimental time. Given that our holographic image is sparse with respect to compactly-supported wavelets, $\ell _{1}$-regularization performs quite well in combination with DB4 even though $K>M$.

### B. Reconstruction and Analysis of Biological Images

We now apply the methodology from Sections II–III to reconstruct and analyze images of biological tissues. We imaged ex vivo samples of mouse oesophagus from healthy controls and carcinogen treated animals with induced oesophageal tumors using the model presented in [5]. We used 3 control mice (6 healthy areas) and 6 mice with tumors (6 distinct lesions). Each sample was segmented into areas of healthy and lesion tissue using the technique of DAPI fluorescence imaging, which was validated in [5]. For more details on preparation of tissue samples, we refer to the supplementary material, Section I-D.

For clarity, we index different areas by $n=1,\ldots,12$, where the first six are healthy and the rest are lesions. Due to the limited field of view of the endoscope ($\sim 200 \mu \mathrm {m}$) relative to the sample size (~2mm), each of the 12 areas produce 6–20 individual images corresponding to different parts of the same sample that may overlay by up to 15%. We thus also introduce index $i$ to denote individual sub-images within a larger area on a given sample, so that each individual sample has index $(n,i)$, $n=1,\ldots,12$, $i=1,\ldots, I_{n}$, for $I_{n}$ in the range 6–20.

**Fig. 8.**
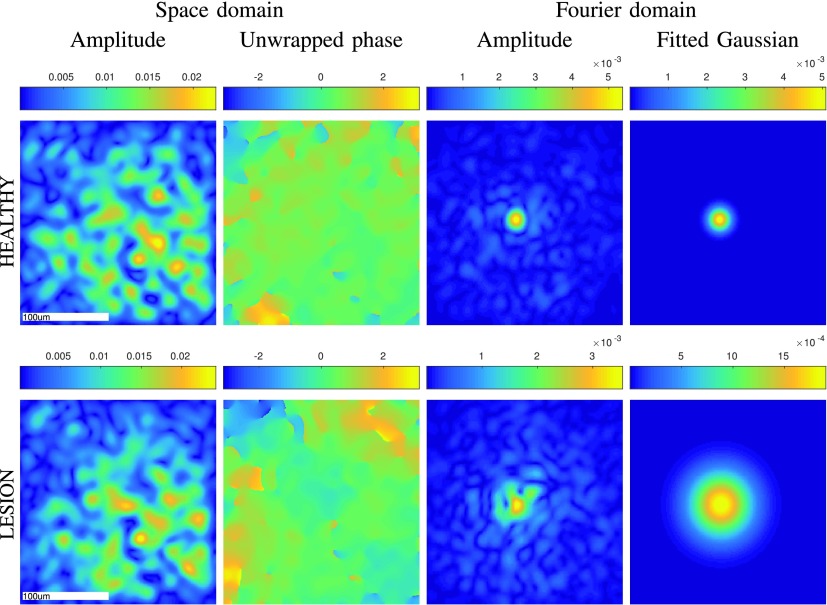
Reconstructed images (horizontal polarization) of healthy and lesion tissue with respect to ${K}=\textsf {400}$ Fourier coefficients by solving the linear system (15) with $\ell _{\textsf {1}}$-regularization and $\lambda =\textsf {0.25}$. The reconstruction time^1^ is around 15s, while the time of the subsequent phase unwrapping and Gaussian-fitting is under 1s.

Fig. 8 shows the reconstruction of the horizontal polarization of one healthy image indexed as (1, 1) and one lesion image indexed as (7, 1), in both the space-domain and the Fourier-domain. Specifically, we reconstruct $K=400$ Fourier coefficients per polarization by solving (15) with $\ell _{1}$-regularization. We then expand these coefficients with respect to Fourier-exponentials to get images in the space-domain, and, with respect to sinc-functions to obtain images in the Fourier-domain. In the space-domain, we show the amplitude and the unwrapped phase of the reconstructed image, where for the unwrapping we used the efficient algorithm from [26]. In the Fourier domain, we show the amplitude of the reconstructed Fourier transform and the corresponding Gaussian fit, where we used the procedure explained in Section III. While the difference between the healthy and the lesion sample is not so apparent from the amplitude and phase in the space-domain, it becomes more pronounced in the Fourier domain; specifically, we observe that the Fourier coefficients decay slower in the lesion than in the healthy tissue, where the decay is quantified by the standard deviation of the fitted Gaussian. For reference, in Fig. 10 we also include the microscopic images of the same healthy and lesion regions as those shown in Fig. 8. Additional images across the data set can be found in the supplementary material, Fig. 17–18.

**Fig. 9.**
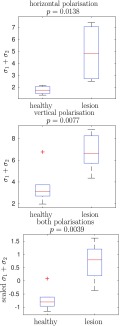
Standard deviation $\sigma _{\textsf {1}}^{({n}{)}}+\sigma _{\textsf {2}}^{({n}{)}}$, ${n}=\textsf {1},\ldots,\textsf {12}$, of a Gaussian fitted to the amplitude of the Fourier transform of 6 healthy and 6 lesion mouse samples. In the bottom box-plot, prior to the t-test, a 2D feature corresponding to two different polarizations is rescaled by the mean and standard deviation of the total of 12 samples.

**Fig. 10.**
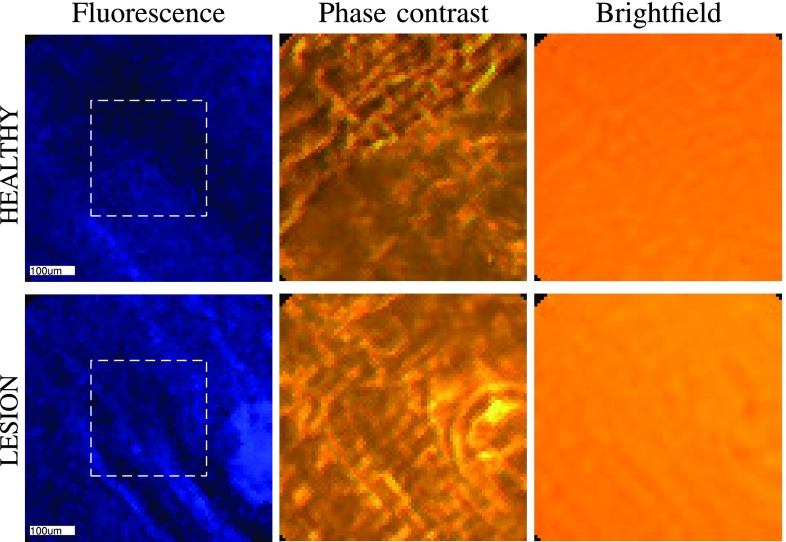
Images obtained by three different microscope modalities: fluorescence, phase contrast and brightfield imaging. The central parts ($\sim \textsf {200}\times \textsf {200} \mu \textsf {m}^{\textsf {2}}$) of the healthy and lesion regions correspond to the images reconstructed via the proposed method in Fig. 8. Fluorescence images with DAPI stain were used to determine the lesion vs. healthy regions. The phase contrast images show that phase information encodes scattering information in lesion areas, while the brightfield images show that under normal ’white light’ used in the conventional endoscopes, features linked with lesions cannot easily be distinguished from healthy tissue.

Finally, in Fig. 9 we perform a statistical test using all samples in the data set, which confirms that the standard deviation of the fitted Gaussians is an informative feature to distinguish between healthy and lesion tissues. In particular, for each individual sample $(n,i)$ we compute $\sigma _{1}^{(n,i)}+\sigma _{2}^{(n,i)}$ of the fitted Gaussian. Then, for each tissue sample $n=1,\ldots,12$, we compute the average $\sigma _{1}^{(n)}+\sigma _{2}^{(n)}:=I_{n}^{-1}\sum _{i=1}^{I_{n}}(\sigma _{1}^{(n,i)}+\sigma _{2}^{(n,i)})$ and group them in a box-plot according to their class label ‘healthy’ or ‘lesion’, for each polarization and for both polarizations combined. We also compute the $p$-value of Welch’s t-test [52], showing the significant difference in the decay of Fourier coefficients between healthy and lesion samples.

We conclude that the degree to which the recovered Fourier coefficients decay, quantified by the standard deviation of the fitted Gaussian, is a feature with a discriminative power, which in the future, in conjunction with a larger data set, could be used to build an automated classifier to distinguish between healthy and lesion samples.

## V. Discussion and Future Research

The main contributions of this paper are two-fold. Firstly, we showed that a Fourier representation of the optical field reflected from a tissue yields a promising diagnostic indicator, using both simulated and experimental real-world data. Secondly, we provided a general reconstruction algorithm that through regularization can stably recover such representation directly from the calibration measurements and the samples of the output field transmitted through a fiber, where the system used for calibration is allowed to be different and thus more efficient than a Fourier basis.

Nevertheless several open problems remain. One such problem relates to learning an ‘optimal’ dictionary (alternative to Fourier) as a means to minimize the classification error between healthy and lesion tissues, which would require a significantly larger number of biological samples to be tested. More importantly, further work is required to enable real-time imaging through fiber endoscopes operating in reflection in realistic clinical settings. Specifically, future research is needed to lift the time-independence assumption present in the kernel of the linear model (1), which in everyday clinical use varies across time with bending and temperature. In practice, the time-independence assumption means that the calibration measurements need to be taken often and under similar bending and temperature conditions as when sampling the output optical field, which is difficult to achieve in realistic clinical deployments unless using a rigid endoscope. The development of a clinically-feasible recovery procedure that accounts for significant fiber changes thus remains an important open problem.

## References

[1] Adcock B., Hansen A., Roman B., and Teschke G., “Chapter four–generalized sampling: stable reconstructions, inverse problems and compressed sensing over the continuum,” Adv. Imag. Electron Phys., vol. 182, pp. 187–279, Feb. 2014.

[2] de Aguiar H. B., Gigan S., and Brasselet S., “Polarization recovery through scattering media,” Sci. Adv., vol. 3, no. 9, p. e1600743, 2017. 28879230 10.1126/sciadv.1600743 PMC5580879

[3] Ahmad I., Ahmad M., Khan K., Ashraf S., Ahmad S., and Ikram M., “*Ex vivo* characterization of normal and adenocarcinoma colon samples by Mueller matrix polarimetry,” J. Biomed. Opt., vol. 20, no. 2, p. 056012, 2015. 10.1117/1.JBO.20.5.056012 26021717

[4] Alali S. and Vitkin I. A., “Polarized light imaging in biomedicine: emerging Mueller matrix methodologies for bulk tissue assessment,” J. Biomed. Opt., vol. 20, no. 6, p. 061104, 2015. 10.1117/1.JBO.20.6.061104 25793658

[5] Alcolea M. P., Greulich P., Wabik A., Frede J., Simons B. D., and Jones P. H., “Differentiation imbalance in single oesophageal progenitor cells causes clonal immortalization and field change,” Nature Cell Biol., vol. 16, no. 2, pp. 612–619, 2014. 10.1038/ncb2963 PMC4085550 24814514

[6] Alexandrov S. A., Hillman T. R., and Sampson D. D., “Spatially resolved Fourier holographic light scattering angular spectroscopy,” Opt. Lett., vol. 30, no. 24, pp. 3305–3307, 2005. 16389813 10.1364/ol.30.003305

[7] Anaparthy R. and Sharma P., “Progression of Barrett oesophagus: Role of endoscopic and histological predictors,” Nature Rev. Gastroenterol. Hepatol., vol. 11, pp. 525–534, 5 2014. 24860927 10.1038/nrgastro.2014.69

[8] Arifler D., Pavlova I., Gillenwater A., and Richards-Kortum R., “Light scattering from collagen fiber networks: Micro-optical properties of normal and neoplastic stroma,” Biophys. J., vol. 92, pp. 3260–3274, 5 2007. 17307834 10.1529/biophysj.106.089839 PMC1852360

[9] Ba C., Palmiere M., Ritt J., and Mertz J., “Dual-modality endomicroscopy with co-registered fluorescence and phase contrast,” Biomed. Opt. Express, vol. 7, no. 9, pp. 3403–3411, 2016. 27699107 10.1364/BOE.7.003403 PMC5030019

[10] Becker S., Bobin J., and Candès E. J., “NESTA: A fast and accurate first-order method for sparse recovery,” SIAM J. Imag. Sci., vol. 4, no. 1, pp. 1–39, 2011.

[11] van den Berg E. and Friedlander M. P., “Probing the Pareto frontier for basis pursuit solutions,” SIAM J. Sci. Comput., vol. 31, no. 2, pp. 890–912, 2008.

[12] van den Berg E. and Friedlander M. P. (2007). SPGL1: A Solver for Large-Scale Sparse Reconstruction. [Online]. Available: http://www.cs.ubc.ca/labs/scl/spgl1

[13] Carpenter J., Eggleton B. J., and Schröeder J., “Maximally efficient imaging through multimode fiber,” in Proc. CLEO, 2014, p. STh1H.3.

[14] Carpenter J., Eggleton B. J., and Schröder J., “}{}$110\times110$ optical mode transfer matrix inversion,” Opt Express, vol. 22, no. 1, pp. 96–101, 2014. 24514970 10.1364/OE.22.000096

[15] Christensen O., An Introduction to Frames and Riesz Bases. Basel, Switzerland: Birkhäuser Verlag, 2002.

[16] Cižmár T. and Dholakia K., “Tunable Bessel light modes: Engineering the axial propagation,” Opt Express, vol. 17, no. 18, pp. 15558–15570, 2009. 19724554 10.1364/OE.17.015558

[17] Cižmár T. and Dholakia K., “Shaping the light transmission through a multimode optical fibre: Complex transformation analysis and applications in biophotonics,” Opt Express, vol. 19, no. 20, pp. 18871–18884, 2011. 21996829 10.1364/OE.19.018871

[18] Cižmár T. and Dholakia K., “Exploiting multimode waveguides for pure fibre-based imaging,” Nature Commun., vol. 3, Aug. 2012, Art. no. 1027. 10.1038/ncomms2024 PMC3432471 22929784

[19] Cohen A., Daubechies I., and Vial P., “Wavelets on the interval and fast wavelet transforms,” Appl. Comput. Harmon. Anal., vol. 1, no. 1, pp. 54–81, 1993.

[20] Doostan A. and Owhadi H., “A non-adapted sparse approximation of PDEs with stochastic inputs,” J. Comput. Phys., vol. 230, no. 8, pp. 3015–3034, Apr. 2011.

[21] Drezek R., Dunn A., and Richards-Kortum R., “Light scattering from cells: Finite-difference time-domain simulations and goniometric measurements,” Appl. Opt, vol. 38, no. 16, pp. 3651–3661, 1999. 18319970 10.1364/ao.38.003651

[22] Drezek R. A., “Light scattering from cervical cells throughout neoplastic progression: Influence of nuclear morphology, DNA content, and chromatin texture,” J. Biomed. Opt., vol. 8, no. 1, pp. 7–16, Jan. 2003. 12542374 10.1117/1.1528950

[23] Gataric M., “Nonuniform generalized sampling,” M.S. thesis, Univ. Cambridge, Cambridge, U.K., 2016, doi: 10.17863/CAM.8433.

[24] Goodman J. W., Statistical Optics. New York, NY, USA: Wiley, 2000.

[25] Gordon G. S. D., “Quantitative phase and polarisation endoscopy for detection of early oesophageal tumourigenesis,” Univ. Cambridge, Cambridge, U.K., Tech. Rep., 2018.

[26] Herráez M. A., Burton D. R., Lalor M. J., and Gdeisat M. A., “Fast two-dimensional phase-unwrapping algorithm based on sorting by reliability following a noncontinuous path,” Appl. Opt., vol. 41, no. 35, pp. 7437–7444, 2002. 12502301 10.1364/ao.41.007437

[27] Jo Y., Jung J., Kim M.-H., Park H., Kang S.-J., and Park Y., “Label-free identification of individual bacteria using Fourier transform light scattering,” Opt Express, vol. 23, no. 12, pp. 15792–15805, 2015. 26193558 10.1364/OE.23.015792

[28] Kim Y. L., “Simultaneous measurement of angular and spectral properties of light scattering for characterization of tissue microarchitecture and its alteration in early precancer,” IEEE J. Sel. Topics Quantum Electron., vol. 9, no. 2, pp. 243–256, Mar. 2003.

[29] Kunnen B., Macdonald C., Doronin A., Jacques S., Eccles M., and Meglinski I., “Application of circularly polarized light for non-invasive diagnosis of cancerous tissues and turbid tissue-like scattering media,” J. Biophoton., vol. 8, pp. 317–323, Apr. 2015. 10.1002/jbio.201400104 25328034

[30] Loterie D., Farahi S., Papadopoulos I., Goy A., Psaltis D., and Moser C., “Digital confocal microscopy through a multimode fiber,” Opt Express, vol. 23, no. 18, pp. 23845–23858, 2015. 26368478 10.1364/OE.23.023845

[31] McClatchy D. M., “Wide-field quantitative imaging of tissue microstructure using sub-diffuse spatial frequency domain imaging,” Optica, vol. 3, no. 6, pp. 613–621, 2016. 27547790 10.1364/OPTICA.3.000613 PMC4989924

[32] Mourant J. R., Johnson T. M., Carpenter S., Guerra A., Aida T., and Freyer J. P., “Polarized angular-dependent spectroscopy of epithelial cells and epithelial cell nuclei to determine the size scale of scattering structures,” J. Biomed. Opt., vol. 7, no. 3, pp. 378–387, 2002. 12175287 10.1117/1.1483317

[33] Pierangelo A., “*Ex-vivo* characterization of human colon cancer by Mueller polarimetric imaging,” Opt Express, vol. 19, no. 2, pp. 1582–1593, 2011. 21263698 10.1364/OE.19.001582

[34] Plöschner M., Tyc T., and Cižmár T., “Seeing through chaos in multimode fibres,” Nature Photon., vol. 9, pp. 529–535, Jul. 2015.

[35] Popoff S. M., Lerosey G., Carminati R., Fink M., Boccara A. C., and Gigan S., “Measuring the transmission matrix in optics: An approach to the study and control of light propagation in disordered media,” Phys. Rev. Lett., vol. 104, p. 100601, Mar. 2010. 20366410 10.1103/PhysRevLett.104.100601

[36] Popoff S., Lerosey G., Fink M., Boccara A. C., and Gigan S., “Image transmission through an opaque material,” Nature Commun., vol. 1, Sep. 2010, Art. no. 81. 10.1038/ncomms1078 20865799

[37] Popoff S. M., Lerosey G., Fink M., Boccara A. C., and Gigan S., “Controlling light through optical disordered media: Transmission matrix approach,” New J. Phys., vol. 13, p. 123021, Dec. 2011.

[38] Pyhtila J. W., Boyer J. D., Chalut K. J., and Wax A., “Fourier-domain angle-resolved low coherence interferometry through an endoscopic fiber bundle for light-scattering spectroscopy,” Opt. Lett., vol. 31, no. 6, pp. 772–774, 2006. 16544619 10.1364/ol.31.000772

[39] Qi J. and Elson D. S., “A high definition Mueller polarimetric endoscope for tissue characterisation,” Sci. Rep., vol. 6, 5 2016, Art. no. 25953. 10.1038/srep25953 PMC4865982 27173145

[40] Rotter S. and Gigan S., “Light fields in complex media: Mesoscopic scattering meets wave control,” Rev. Mod. Phys., vol. 89, p. 015005, Mar. 2017.

[41] Shen Y., Liu Y., Ma C., and Wang L. V., “Focusing light through biological tissue and tissue-mimicking phantoms up to 9.6 cm in thickness with digital optical phase conjugation,” J. Biomed. Opt., vol. 21, no. 8, p. 85001, 2016. 27533439 10.1117/1.JBO.21.8.085001 PMC4982119

[42] Snyder A. W. and Love J., Optical Waveguide Theory. New York, NY, USA: Chapman & Hall, 1983.

[43] Sridharan S., Macias V., Tangella K., Kajdacsy-Balla A., and Popescu G., “Prediction of prostate cancer recurrence using quantitative phase imaging,” Sci. Rep., vol. 5, p. 9976, 5 2015. 25975368 10.1038/srep09976 PMC4432311

[44] Su J.-W., “Precancerous esophageal epithelia are associated with significantly increased scattering coefficients,” Biomed. Opt. Express, vol. 6, no. 10, pp. 3795–3805, 2015. 26504630 10.1364/BOE.6.003795 PMC4605039

[45] Thompson A. J., Paterson C., Neil M. A. A., Dunsby C., and French P. M. W., “Adaptive phase compensation for ultracompact laser scanning endomicroscopy,” Opt. Lett., vol. 36, no. 9, pp. 1707–1709, 2011. 21540976 10.1364/OL.36.001707

[46] Wang H.-D., Niu C. H., Yang Q., and Badea I., “Study on protein conformation and adsorption behaviors in nanodiamond particle–protein complexes,” Nanotechnology, vol. 22, no. 14, p. 145703, 2011. 21346296 10.1088/0957-4484/22/14/145703

[47] Wang Z., Tangella K., Balla A., and Popescu G., “Tissue refractive index as marker of disease,” J. Biomed. Opt., vol. 16, no. 11, p. 116017, Nov. 2011. 22112122 10.1117/1.3656732 PMC3223513

[48] Wang J., “Tribological, anti-corrosive properties and biocompatibility of the micro- and nano-crystalline diamond coated Ti6Al4V,” Surf. Coat. Technol., vol. 258, pp. 1032–1038, Nov. 2014.

[49] Warren S. C., “Adaptive multiphoton endomicroscopy through a dynamically deformed multicore optical fiber using proximal detection,” Opt Express, vol. 24, no. 19, pp. 21474–21484, 2016. 27661887 10.1364/OE.24.021474

[50] Waterhouse D. J., Fitzpatrick C. R. M., di Pietro M., and Bohndiek S. E., “Emerging optical methods for endoscopic surveillance of Barrett’s oesophagus,” LANCET Gastroenterol. Hepatol., vol. 3, no. 5, pp. 349–362, 2018. 29644977 10.1016/S2468-1253(18)30030-X

[51] Wax A. and Chalut K. J., “Nuclear morphology measurements with angle-resolved low coherence interferometry for application to cell biology and early cancer detection,” Anal. Cellular Pathol., vol. 34, no. 5, pp. 207–222, 2011. 10.3233/ACP-2011-0017 PMC3641557 21988885

[52] Welch B. L., “The generalization of ‘student’s’ problem when several different population variances are involved,” Biometrika, vol. 34, pp. 28–35, Jan. 1947. 20287819 10.1093/biomet/34.1-2.28

